# Accurate Automatic Detection of Densely Distributed Cell Nuclei in 3D Space

**DOI:** 10.1371/journal.pcbi.1004970

**Published:** 2016-06-06

**Authors:** Yu Toyoshima, Terumasa Tokunaga, Osamu Hirose, Manami Kanamori, Takayuki Teramoto, Moon Sun Jang, Sayuri Kuge, Takeshi Ishihara, Ryo Yoshida, Yuichi Iino

**Affiliations:** 1 Department of Biological Sciences, Graduate School of Science, The University of Tokyo, Bunkyo-ku, Tokyo, Japan; 2 Department of Systems Design and Informatics, Faculty of Computer Science and Systems Engineering, Kyushu Institute of Technology, Iizuka-shi, Fukuoka, Japan; 3 The Institute of Statistical Mathematics, Research Organization of Information and Systems, Tachikawa, Tokyo, Japan; 4 Faculty of Electrical and Computer Engineering, Institute of Science and Engineering, Kanazawa University, Kakuma, Kanazawa, Japan; 5 Department of Biology, Faculty of Sciences, Kyushu University, Higashi-ku, Fukuoka, Japan; 6 CREST, Japan Science and Technology Corporation, Bunkyo-ku, Tokyo, Japan; University College London, UNITED KINGDOM

## Abstract

To measure the activity of neurons using whole-brain activity imaging, precise detection of each neuron or its nucleus is required. In the head region of the nematode *C*. *elegans*, the neuronal cell bodies are distributed densely in three-dimensional (3D) space. However, no existing computational methods of image analysis can separate them with sufficient accuracy. Here we propose a highly accurate segmentation method based on the curvatures of the iso-intensity surfaces. To obtain accurate positions of nuclei, we also developed a new procedure for least squares fitting with a Gaussian mixture model. Combining these methods enables accurate detection of densely distributed cell nuclei in a 3D space. The proposed method was implemented as a graphical user interface program that allows visualization and correction of the results of automatic detection. Additionally, the proposed method was applied to time-lapse 3D calcium imaging data, and most of the nuclei in the images were successfully tracked and measured.

## Author Summary

To reach the ultimate goal of neuroscience to understanding how each neuron functions in the brain, whole-brain activity imaging techniques with single-cell resolution have been intensively developed. There are many neurons in the whole-brain images and manual detection of the neurons is very time-consuming. However, the neurons are often packed densely in the 3D space and existing automatic methods fail to correctly split the clumps. In fact, in previous reports of whole-brain activity imaging of *C*. *elegans*, the number of detected neurons were less than expected. Such scarcity may be a cause of measurement errors and misidentification of neuron classes. Here we developed a highly accurate automatic cell detection method for densely-packed cells. The proposed method successfully detected almost all neurons in whole-brain images of the nematode. Our method can be used to track multi-objects and enables automatic measurements of the neuronal activities from whole-brain activity imaging data. We also developed a visualization and correction tool that is helpful for experimenters. Additionally, the proposed method can be a fundamental technique for other applications such as making wiring diagram of neurons or establishing a cell lineage in embryonic development. Thus our framework supports effective and accurate bio-image analyses.

## Introduction

The animal brain is the most complex information processing system in living organisms. To elucidate how real nervous systems perform computations is one of the fundamental goals of neuroscience and systems biology. The wiring information for neural circuits and visualization of their activity at cellular resolution are required for achieving this goal. Advances in microscopy techniques in recent years have enabled whole-brain activity imaging of small animals at cellular resolution [1–4]. The wiring information of all the neurons in the mouse brain can be obtained using recently developed brain-transparentization techniques [5–9].

Detection of neurons from microscopy images is necessary for optical measurements of neuronal activity or for obtaining wiring information. Because there are many neurons in the images, methods of automatic neuron detection, rather than manual selection of ROIs (regions of interest), are required and several such methods have been proposed [10,11]. Detection of cells that are distributed in three-dimensional (3D) space is also important in other fields of biology such as embryonic development studies [12–17].

In these methods, cell nuclei are often labeled by fluorescent probes and used as a marker of a cell. To identify nuclei in such images, the basic method is blob detection, which for example consists of local peak detection followed by watershed segmentation. If the cells are sparsely distributed, blob detection methods are powerful techniques for nucleus detection. However, if two or more cells are close to each other, the blobs are fused, and some cells will be overlooked. These false negatives may be trivial for the statistics of the cells but may strongly affect individual measurements such as those of neuronal activity. Overlooking some nuclei should be avoided when subsequent analyses assume that all the cells were detected, for example, when making wiring diagram of neurons or establishing a cell lineage in embryonic development. Therefore, correct detection of all nuclei from images without false negatives is a fundamental problem in the field of bio-image informatics.

Although many efforts have been made to develop methods that avoid such false negatives, these methods seem to insufficiently overcome the problem. In the head region of *Caenorhabditis elegans*, for example, the neuronal nuclei are densely packed and existing methods produce many false negatives, as shown below. Actually, in the studies of whole-brain activity imaging of *C*. *elegans* reported so far, the local peak detection method that can overlook many nuclei was employed [3,18], or the nuclei were manually detected [19,20]. Highly accurate automatic nucleus detection methods should be developed in order to improve the efficiency and accuracy of such image analysis.

Here we propose a highly accurate automatic nucleus detection method for densely distributed cell nuclei in 3D space. The proposed method is based on newly developed clump splitting method suitable for 3D images and improves the detection of all nuclei in 3D images of neurons of nematodes. A combination of this approach with a Gaussian mixture fitting algorithm yields highly accurate locations of densely packed nuclei and enables automatic tracking and measuring of these nuclei. The performance of the proposed method is demonstrated by using various images of densely-packed head neurons of nematodes which was obtained by various types of microscopes.

## Results

### Overlooking of nuclei by blob detection

**Fig 1:**
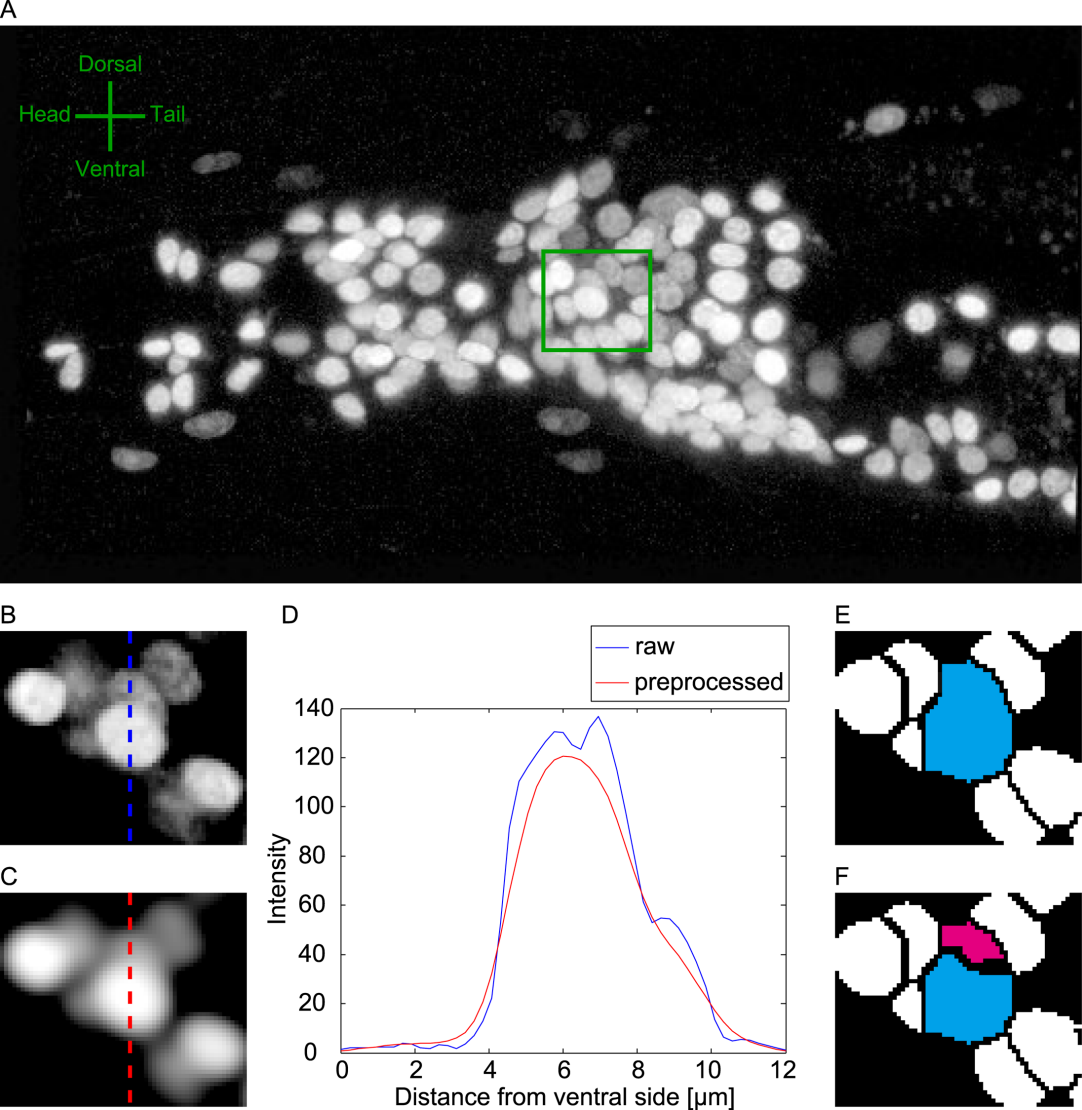
Overlooking of nuclei by conventional blob detection. **(A)** Example of 3D image of neuronal nuclei in the head region of a worm (Data 1, see Methods). Image is displayed as max projection along *x*_3_ axis. Note that all of the raw images in this paper is displayed in logarithmic scale in order to visualize dark nuclei. **(B)** Enlarged view of green box in (A). A particular slice along the *x*_3_ axis is displayed. **(C)** Preprocessed image of the image in (B). **(D)** Intensities on the dotted lines in (B) and (C). **(E)** Results of watershed segmentation (step 2 of the proposed method). The region of the brighter nucleus is shown in cyan. **(F)** Results of segmentation by the proposed method (step 3 of the proposed method). The region of the darker nucleus is shown in magenta.

In this study, we focused on the head neurons of the soil nematode *C*. *elegans*, which constitute the major neuronal ensemble of this animal [21]. All the neuronal nuclei in a worm of strain JN2100 were visualized by the red fluorescent protein mCherry. The head region of the worm was imaged by a confocal microscope, and we obtained 3D images of 12 animals (Data 1, Fig 1A). The shape of the nuclei was roughly ellipsoidal (Fig 1B). The fluorescence intensity increased toward the centers of the nuclei (Fig 1D). The typical half-radius of the nuclei was about 1.10 μm (S1 Fig). The distance to the nearest neighboring nucleus was 4.30 ± 2.13 μm (mean and standard deviation, S1 Fig), suggesting that the neurons are densely distributed in 3D space. The mean fluorescence intensities differed among neurons by one order of magnitude (S1 Fig), making it difficult to detect a darker nucleus near a bright nucleus.

We first applied conventional blob detection techniques to the 3D image (Fig 1C–1E). Salt-and-pepper noise and background intensities were removed from the image. The image was smoothed to avoid over-segmentation (Fig 1C and 1D). Local intensity peaks in the preprocessed image were detected and used as seeds for 3D seeded grayscale watershed segmentation. Each segmented region was regarded as a nucleus (Fig 1E). We found that dark nuclei in high-density regions often escaped detection. If the dark nucleus was adjacent to a bright nucleus, the fluorescence of the bright nucleus overlapped that of the dark one, and the local intensity peak in the dark nucleus was masked (Fig 1D). As a result, the seed for the dark nucleus was lost, and the dark nucleus fused with the bright nucleus (Fig 1E). The rate of false-negative nuclei was 18.9%. In contrast, our proposed method successfully detected and segmented the dark nuclei (Fig 1F).

### Using areas of negative curvature of iso-intensity surfaces for clump splitting in 3D images

**Fig 2:**
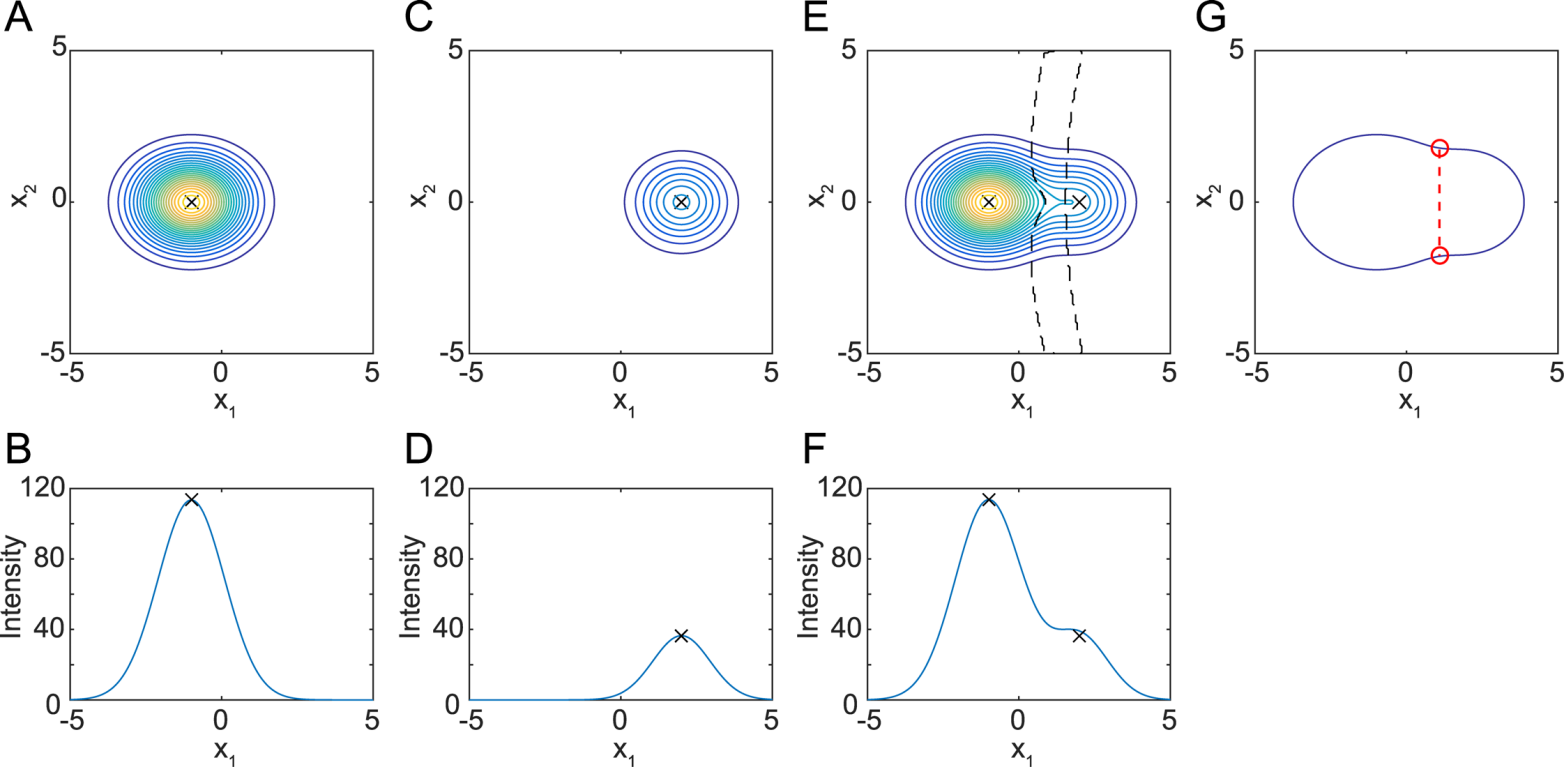
Fluorescence intensity distribution of nuclei approximated by Gaussian distribution. (**A, B**) Example of fluorescence intensity distribution of a bright nucleus approximated by Gaussian distribution *f*_1_. *π*_1_ = 120, *μ*_1_ = (−1,0,0), *Σ*_1_ = (diag(1.10,0.89,1.35))^2^. (**C**, **D**) Example of fluorescence intensity distribution of a dark nucleus approximated by Gaussian distribution *f*_2_. *π*_2_ = 40, *μ*_2_ = (2,0,0), *Σ*_2_ = (diag(1.10,0.89,1.35) × 0.9)^2^. (**E**, **F**) Fluorescence intensity distribution when a dark nucleus is near a bright nucleus, approximated by a mixture of distributions *f*_1_ and *f*_2_. Area enclosed by black broken lines in E displays an area of negative curvature. (**A**, **C**, **E**) Iso-intensity contour lines of trivariate Gaussian distribution at *x*_3_ = 1. (**B**, **D**, **F**) Plot of intensity at (*x*_2_,*x*_3_) = (0,1). Black crosses indicate peak positions. (**G**) Outline of the fused blob and typical result of 2D clump splitting method. Red points indicate concave points. Red dashed line indicates the border line.

The shapes of the nuclei are roughly ellipsoidal, and the fluorescence intensity increased toward the centers of the nuclei, suggesting that the intensity of nuclei can be approximated by a mixture of trivariate Gaussian distributions. The intensities *f*_*k*_ of the *k*-th Gaussian distribution *g*_*k*_ at voxel position $x\in R^{3}$ can be written as

$$f_{k}(x)=\pi_{k}g_{k}(x\;|\;\mu_{k},\Sigma_{k})=\pi_{k}\exp(-\frac{1}{2}{\left(x-\mu_{k}\right)}^{\text{T}}\Sigma_{k}^{-1}(x-\mu_{k})),$$

where *μ*_*k*_ and *Σ*_*k*_ are the mean vector and covariance matrix of *g*_*k*_, respectively, and *π*_*k*_ is an intensity scaling factor. To explain the effect on the curvature, typical bright and dark nuclei were approximated by the Gaussian distribution and are shown in Fig 2 as iso-intensity contour lines (Fig 2A, 2C and 2E) and plots of the intensity along the cross section (Fig 2B, 2D and 2F). When a bright nucleus was near a dark nucleus, the peak intensity of the dark nucleus merged with the tail of the fluorescence intensity distribution of the bright nucleus and no longer formed a peak.

These false negatives can be avoided by using methods for dividing a close pair of objects, or clump splitting. Such methods have been developed for correct detection of objects in two-dimensional (2D) images [22–26]. These methods focus on the concavity of the outline of a blob. The concavity was calculated based on one of or a combination of various measurements such as angle [25], area [27], curvature [26], and distance measurements [24] of the outline. In these methods, after binarization of the image, concavity was obtained for each point on the outline. Then the concave points were determined as the local peaks of the concavity. After determination of concave points, a line connecting a pair of concave points is regarded as the boundary between the objects. When we regard the outermost contour line in Fig 2E as the outline of the fused blob (Fig 2G), the conventional 2D clump splitting method can be easily applied and two concave points are detected from the fused blob (Fig 2G, red circles). The blob was divided into two parts by a border line connecting the two points, and the dark nucleus was detected.

In the ideal case in Fig 2E, we obtained necessary and sufficient number of concave points. In real images, however, we might obtain too many concave points because outlines often contain noise and are not smooth. However, the number of concave points to choose is unknown because it is hard to know how many nuclei are included in a blob in a real image. Further, it is not obvious how to find the correct combinations of concave points to be linked if a blob contains three or more objects. In addition, for 3D images, the concepts of border lines that connect two concave points cannot be naturally expanded to three dimensions, because now we need some extra processes such as connecting groups of concave points in order to form border surfaces. Even if we regard a 3D image as a stack of 2D images, it is hard to split objects fused in the z direction (direction of the stacks) [11,27].

Here we introduce a concept of areas of concavity instead of concave points (i.e. local peak of concavity). Hereafter we use curvature as a measure of concavity and focus on areas of negative curvature for simplicity and clarity, but other measures such as angle, area, and distance from convex hull may be applicable. Furthermore we used the iso-intensity contour lines inside the object in addition to the outline of the object. Near the concave points in Fig 2E, the iso-intensity contour lines have negative curvature; i.e., they curve in the direction of low intensities. Negative curvature may be a landmark of the border line because a single Gaussian distribution has positive curvature everywhere. Actually, the voxels at which an iso-intensity contour line has negative curvature were between two Gaussian distributions (Fig 2E, area between the broken lines). Once these voxels are removed from the blob, detection of two nuclei should be straightforward.

This approach is different from the classic clump splitting methods in two respects; focusing on area rather than local peak of concavity (concave points), and using iso-intensity contour lines in addition to the outline. These differences eliminate the need for determining how many concave points should be chosen and for obtaining correct combinations of the concave points because the area of negative curvature will cover the border lines. Therefore we can use the approach even if a blob contains three or more objects. In addition, this approach is robust to noise because it does not depend on a single contour line. Furthermore, this approach can be expanded to 3D images naturally because the 3D area (i.e. voxels) of negative curvatures will cover the border surfaces of the 3D objects. Iso-intensity contour lines in 2D images are parts of iso-intensity contour surfaces in three dimensions. A point on an iso-intensity surface has two principal curvatures, which can be calculated from the intensities of surrounding voxels (S2 Text) [28]. The smaller of the two principal curvatures is positive at any point in a single Gaussian distribution but is negative around the border of two Gaussian distributions. Therefore, once voxels that have negative curvature are removed from the blob, two or more nuclei should be detected easily in 3D images. Thus our approach solves the above problems of the classic clump splitting methods.

### Clump splitting in real 3D images

**Fig 3:**
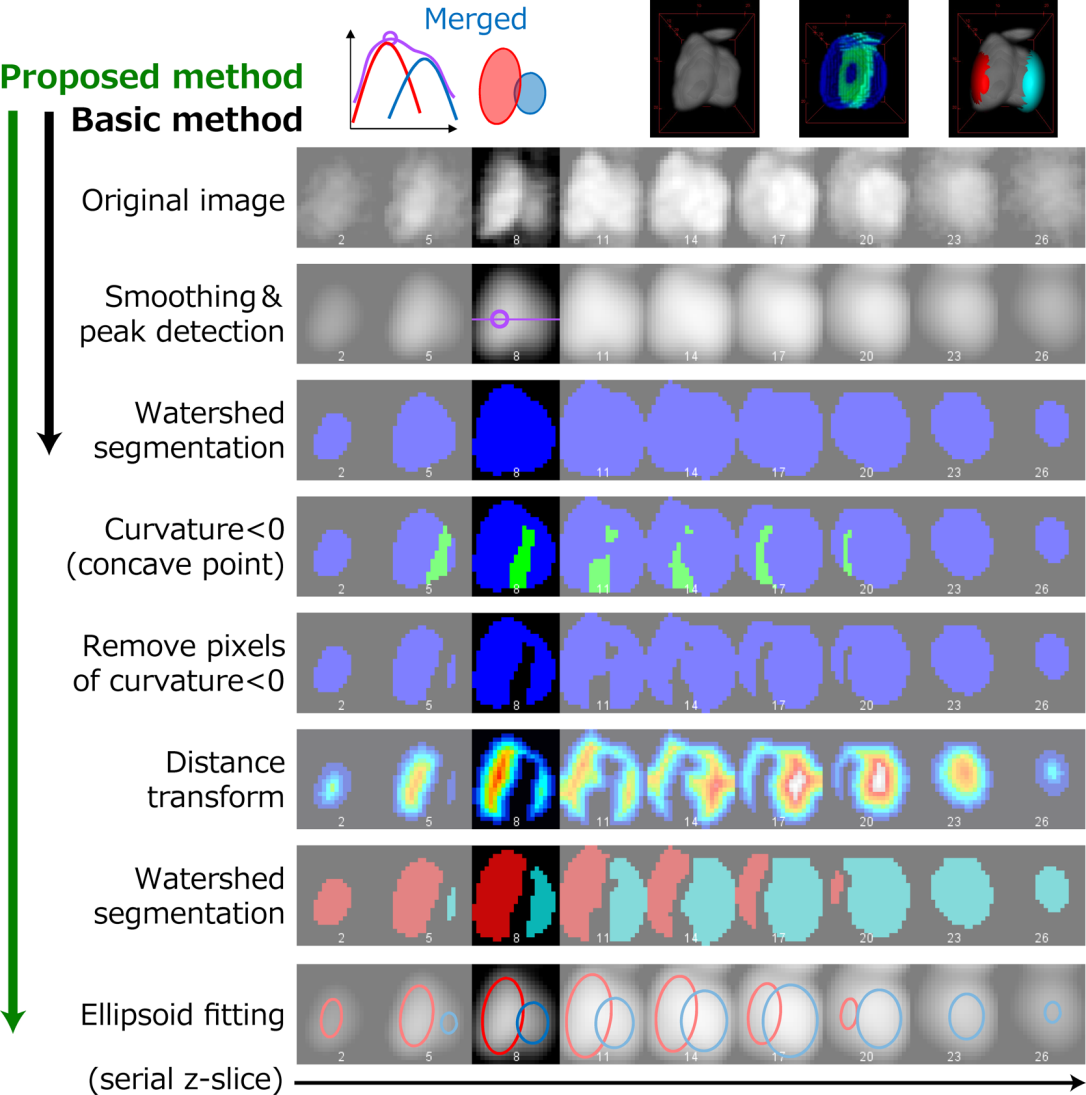
Illustration of the proposed method. Results of each process are shown in each row. Columns show images at different *x*_3_ positions (focal planes).

We applied the above approach to real 3D images (Fig 3). The original images were processed by denoising, background removal, and smoothing to obtain the preprocessed images. The peak detection algorithm could find only a peak from the bright nucleus, and the blob obtained by watershed segmentation contained both nuclei. The principal curvatures of the iso-intensity surface were calculated from the preprocessed image. There were voxels of negative curvature in the area between two nuclei, but the area did not divide the two nuclei completely. The voxels of negative curvature were removed from the blob, and the blob was distance-transformed; these procedures were followed by 3D watershed segmentation. Thus, the two nuclei were separated, and the dark nucleus was successfully detected.

After voxels of negative curvature were removed from the blobs, the size of blobs obtained by the second watershed segmentation tended to be smaller than real nuclei, and the distances between the blobs tended to be larger. To obtain the precise positions and sizes of the nuclei, least squares fitting with a Gaussian mixture was applied to the entire 3D image using a newly developed method (see Methods). The number of Gaussian distributions and the initial values of the centers of the distributions were derived from the above results.

Repeated application of watershed segmentation may increase over-segmentation. If the distance between two fitted Gaussian distributions is too small, the two distributions may represent the same nuclei. In this case, one of the two distributions was removed to avoid over-segmentation, and the fitting procedure was repeated with a single Gaussian distribution.

**Fig 4:**
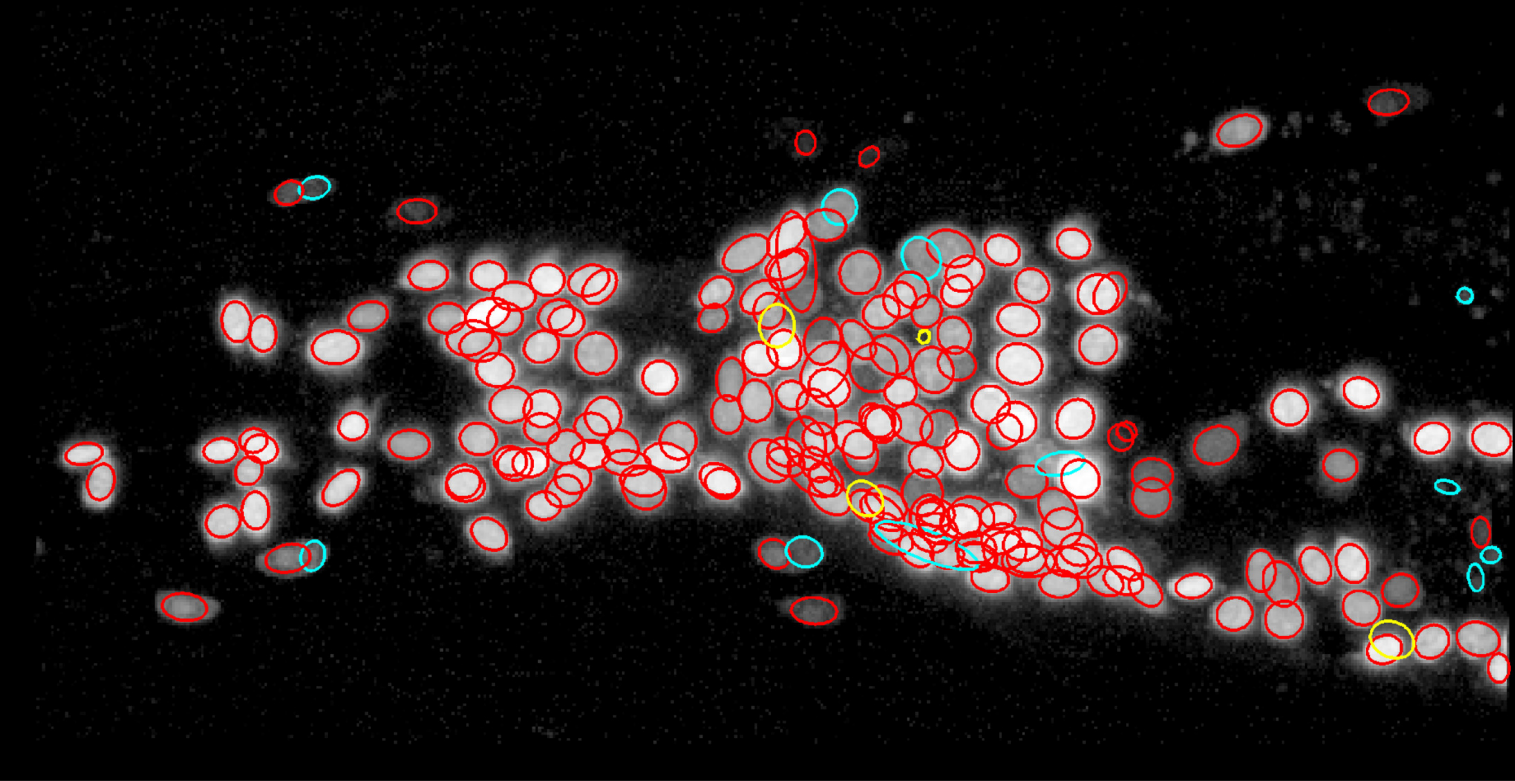
Nuclei detected by the proposed method. True positives, false positives, and false negatives are shown as red, cyan, and yellow ellipses, respectively. Original image is the same as Fig 1A.

The proposed method detected 194 out of 198 nuclei in the 3D image (Fig 4). Among the four overlooked nuclei, the intensities of two of them were too low to be detected. The other two had moderate intensities but were adjacent to brighter nuclei. In these cases, curvature-based clump splitting successfully split the two nuclei. However, deviations of the brighter nuclei from Gaussian distributions disrupted the fitting of the Gaussian distributions and resulted in misplacement of the Gaussian distributions for the darker nuclei, which were instead fitted to the brighter nuclei. On the other hand, the proposed method returned 11 false positives. Two of them resulted from the misplacement of the Gaussian distribution for the darker nuclei described above. Four of them were not neuronal nuclei but were fluorescence foci intrinsic to the gut. Three of them were the result of over-segmentation of complex-shaped non-neuronal nuclei. One of them was mislocalized fluorescence in the cytosol. The last one was the result of over-segmentation of a large nucleus that was fitted with two Gaussian distributions separated by a distance larger than the cutoff distance.

### Comparison with other segmentation methods

**Fig 5:**
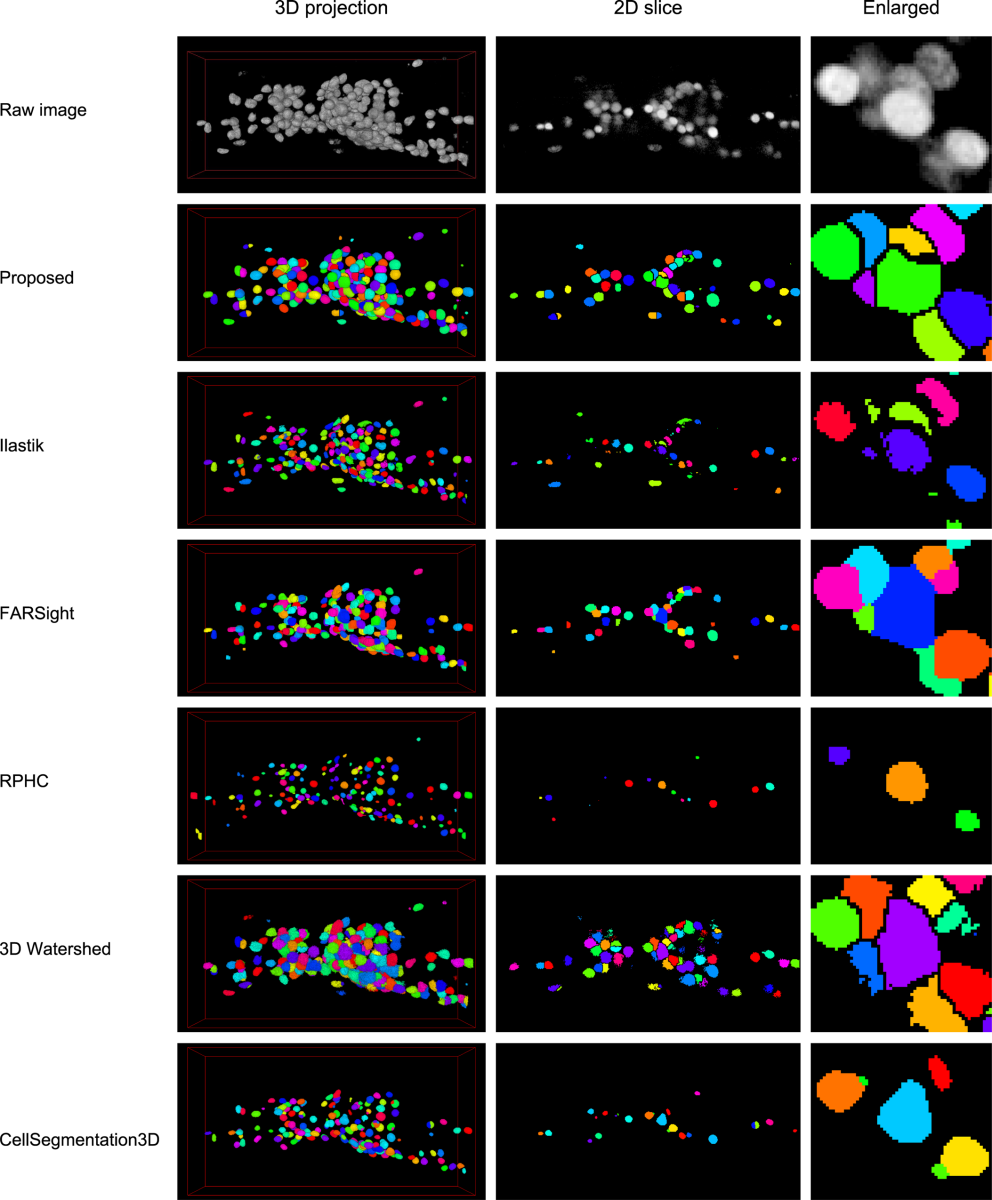
Segmentation results of proposed and previously published methods. Each row shows the original image or the segmentation result of the indicated method. Left column shows the three dimensional projection of the result. Middle column shows the two-dimensional section of the result. Right column shows the enlarged view of the image in the middle column. Each segmented region is assigned a different color.

We compared the performance of the proposed method with five previously published methods for nucleus segmentation (Fig 5 and Table 1). Ilastik [29] is based on machine learning techniques and uses image features such as Laplacian of Gaussian. FARSight [30] is based on graph cut techniques. RPHC [1] was designed for multi-object tracking problems such as whole-brain activity imaging of *C*. *elegans* and uses a numerical optimization-based peak detection technique for object detection. 3D watershed plugin in ImageJ [31] consists of local peak detection and seeded watershed. This method is almost the same as the conventional blob detection method used in our proposed method. CellSegmentation3D [32] uses gradient flow tracking techniques and was developed for clump splitting. This method has been used in the study of automated nucleus detection and annotation in 3D images of adult *C*. *elegans* [33]. We applied these six methods to 12 animals in Data 1 (Fig 5) and obtained the performance indices (Table 1, see Methods). The parameters of each method were optimized for the dataset.

**Table 1:** Performance of proposed and the state-of-the-art methods.

	Proposed	Ilastik	FARSight	RPHC	3D watershed	CellSegmentation3D
TP	184.92	169.67	155.25	141.67	161.17	152.33
FP	9.42	5.08	3.92	11.50	22.08	40.33
FN	6.00	21.25	35.67	49.25	29.75	38.58
Found	194.33	174.75	159.17	153.17	183.25	192.67
TP rate	0.9686	0.8885	0.8133	0.7419	0.8440	0.7981
FP rate	0.0493	0.0265	0.0205	0.0602	0.1158	0.2115
FN rate	0.0314	0.1115	0.1867	0.2581	0.1560	0.2019
F-measure	0.9602	0.9277	0.8865	0.8232	0.8615	0.7943
Accuracy	0.9239	0.8657	0.7969	0.7001	0.7572	0.6597
Time (sec)	202.60	240.75	14.73	537.41	40.98	352.70

The means of 12 animals are shown. TP, FP and FN mean True Positive, False Positive and False Negative, respectively. The mean of the Grand Truth (GT) is 190.92. The details of performance indices are described in Methods section.

The 3D images in the dataset contains 190.92 nuclei on average, based on manual counting. The proposed method found 96.9% of the nuclei and the false negative rate was 3.1%, whereas the false negative rate of the other methods were 11.2% or more. The false positive rate of the proposed method was 4.9% and that of the other methods ranged from 2.1% to 21.2%. The proposed method shows the best performance with both of the well-established indices, F-measure [12] and Accuracy [34], because of the very low false negative rate and modest false positive rate.

It should be noted that all of the compared methods overlooked more than 10% of nuclei in our dataset. The reason for this was suggested by the segmentation results, in which almost all of these methods failed to detect the dark nuclei near the bright nuclei and fused them (Fig 5, right column). These results suggest that all the compared methods have difficulty in handling 3D images with either large variance of object intensity or dense packing of objects, or both (S1 Fig).

These results clearly indicate that our proposed method detects densely distributed cell nuclei in 3D space with highest accuracy. Very low false negative rate is the most significant improvement of the proposed method from the other methods, suggesting that the proposed method will improve efficiency and accuracy of image analysis steps drastically.

### GUI for visualization and error correction

Because none of the computational image analysis methods is perfect, experimenters should be able to correct any errors they find. Therefore, a user-friendly graphical user interface (GUI) for visualization and correction of the results is required. We developed a GUI called RoiEdit3D for visualizing the result of the proposed method and correcting it manually (S2 Fig). Because RoiEdit3D is based on ImageJ/Fiji [35,36] in MATLAB through Miji [37], experimenters can use the familiar interface and tools of ImageJ directly. Developers can extend the functionality using a favorite framework chosen from various options such as ImageJ macros, Java, MATLAB scripts, and C++ languages. Interface with downstream analyses should be straightforward because the corrected results are saved in the standard MATLAB data format and can be exported to Microsoft Excel.

Three-dimensional images are shown as trihedral figures using the customized Orthogonal View plugin in ImageJ (S2 Fig). Fitted Gaussian distributions are shown as ellipsoidal regions of interest (ROIs) in each view. The parameters of the Gaussian distributions are shown in the Customized ROI Manager window in tabular form. The Customized ROI Manager and trihedral figures are linked, and selected ROIs are highlighted in both windows. When the parameters of the distributions or the names of nuclei are changed in the Customized ROI Manager window, the corresponding ROIs in the trihedral figures are updated immediately. Least squares fitting with a Gaussian mixture can be applied after ROIs are manually removed or added.

### Application for whole-brain activity imaging

**Fig 6:**
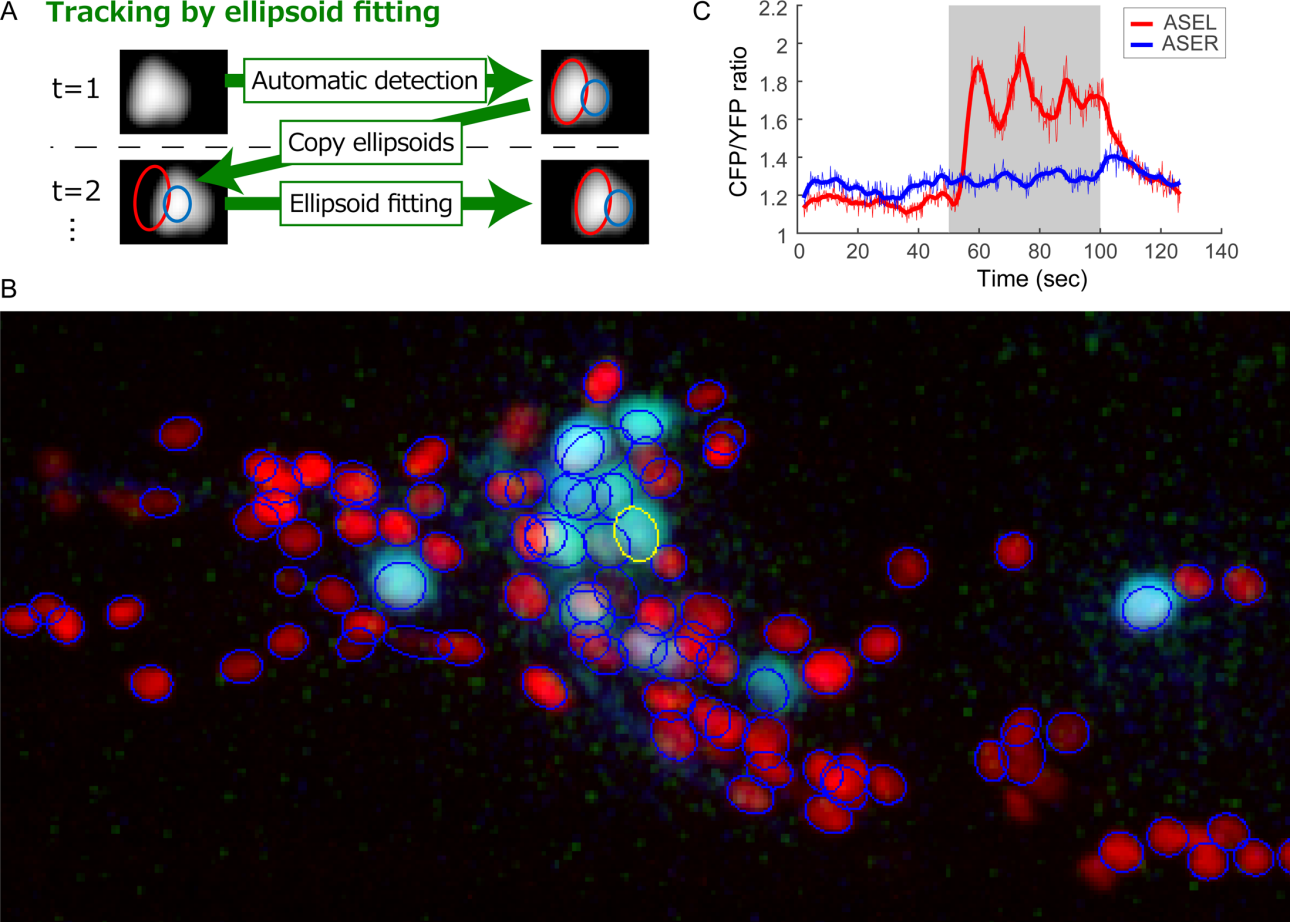
Methods and results of tracking using RoiEdit3D. **(A)** Schematic illustration of the tracking method. **(B)** Part of the data used for tracking (Data 2). Three-dimensional image of the first time point is shown as the max projected image of the right half of the animal. Neuronal nucleus marker is shown in red, and calcium indicator is shown in light blue. ROI of ASER is shown as a yellow ellipse. Other ROIs appear as blue ellipses. **(C)** Time course of ASER response obtained by the calcium indicator. Gray area indicates the stimulation period.

RoiEdit3D can be used for multi-object tracking. The fitted Gaussian mixture at a time point is used as an initial value for the mixture at the next time point, and a fitting procedure is executed (Fig 6A). Additionally, the intensities of nuclei can be obtained as parameters of the fitted Gaussian distributions.

We tried to track and measure the fluorescence intensity of nuclei in real time-lapse 3D images (Data2). The animal in the image expressed a calcium indicator, so neural activity during stimulation with the sensory stimulus, sodium chloride, could be measured as changes in the fluorescent intensity. The proposed nucleus detection method was applied to the first time point in the image and found 194 nuclei out of 198 nuclei. Seventeen false positives and four false negatives were corrected manually using RoiEdit3D. Then the nuclei in the time-lapse 3D image were tracked by the proposed method. Most of the nuclei were successfully tracked. One or more tracking errors occurred in 27 nuclei during 591 frames, and the success rate was 86.4%, which is comparable to that in the previous work [1]. The tracking process takes 19.83 sec per frame (total 3.25 hr).

The ASER gustatory neuron was successfully identified and tracked in the time-lapse 3D image by the proposed method (Fig 6B). The ASER neuron reportedly responds to changes in the sodium chloride concentration [38,39]. We identified a similar response of the ASER neuron using the proposed method (Fig 6C). This result indicates that the proposed method can be used for multi-object tracking and measuring, which is an essential function for whole-brain activity imaging.

Furthermore the proposed method was utilized to measure the fluorescence intensity of nuclei in time-lapse 2D images (Data 3). The proposed nucleus detection method was applied to the image for the first time point (S3 Fig). Data 3 does not contain images of a highly-localized nuclear marker, and therefore the images of calcium indicator that was weakly localized to the nuclei were used instead. The proposed method found 7 nuclei out of 9 nuclei. Six false positives and two false negatives were corrected manually using RoiEdit3D. Then the nuclei were tracked by the proposed method. All of the nuclei were successfully tracked during 241 time frames. The ASER neuron was successfully identified and tracked in the 2D images. The response of the ASER neuron in the 2D images (S3 Fig) is similar to that in the 3D images. This result indicates that the proposed method can be used for multi-object tracking and measuring of 2D images as well as 3D images.

## Discussion

In this article, we proposed a method that accurately detects neuronal nuclei densely distributed in 3D space. Our GUI enables visualization and manual correction of the results of automatic detection of nuclei from 3D images as well as 2D images. Additionally, our GUI successfully tracked and measured multiple objects in time-lapse 2D and 3D images. Thus, the proposed method can be used as a comprehensive tool for analysis of neuronal activity, including whole-brain activity imaging.

Although the microscopy methods for whole-brain activity imaging of *C*. *elegans* have been intensively developed in recent years [3,18–20], computational image analysis methods were underdeveloped. In these works, the neuronal nuclei in the whole-brain activity imaging data were detected either manually or automatically by peak detection. Manual detection is most reliable but time- and labor-consuming, whereas the accuracy of the automatic peak detection is relatively low because of overlooking dark nuclei near bright nuclei. Our proposed method will reduce the difficulty and improve the accuracy. Furthermore, the numbers of the neuronal nuclei found or tracked in these four works were less than the real number of neuronal nuclei [3,18–21]. The scarcity may be due not only to the experimental limitations such as fluctuation of fluorescent protein expression or low image resolution, but also to the limitations of the image analysis methods that may overlook nuclei. The proposed method can detect almost all the nuclei in our whole-brain activity imaging data (Fig 6), suggesting that the proposed method can avoid errors that may be caused by overlooking nuclei, such as erroneous measurements of neural activities and misidentifications of neuron classes. Thus, our method will be highly useful for the purpose.

Peng and colleagues have intensively developed the computational methods for automatic annotation of cell nuclei in *C*. *elegans* [33,40,41]. Although their methods successfully annotate cells in many tissue such as body wall muscles and intestine, the methods seem not to be applicable to annotations of head neurons in adult worms, which is highly desired in the field of whole-brain activity imaging [20]. They pointed out that the positions of neuronal nuclei in adult worms are highly variable [33] and this may be one of the reasons for the difficulty. The accuracy of detection and segmentation of neuronal nuclei may be another reasons because CellSegmentation3D that was incorporated in their latest annotation framework [33] shows compromised performance in our dataset (Table 1, Fig 5). Our proposed method improves the accuracy of neuronal nucleus detection and will promote developing the automatic annotation methods for the neurons. It is noteworthy that the method of simultaneous detection and annotation of cells [41] is unique and useful in the studies of *C*. *elegans*. Because the method assigns the positions of reference to the sample image directly and avoid the detection step, the method find cells without overlooking under some conditions, but would not work correctly under the large variation of the numbers or the relative positions of the nuclei, both of which are observed in our dataset.

The optimal method for accurate detection of nuclei will vary depending on the characteristics of the nuclei. Many conditions such as the visualization method, shape, and distribution of nuclei will affect these characteristics. In our case, the distributions of the fluorescence intensity of nuclei were similar to Gaussian distributions; thus, we developed an optimal method for such cases. Even if an original image does not have these characteristics, some preprocessing steps such as applying a Gaussian smoothing filter may enable application of our method to the image.

Although choosing the optimal method and tuning its parameters might be more work than manual identification, the automatic detection method would improve subjectivity and effectivity. In the field of biology, it is often the case that hundreds or thousands of animals should be analyzed equally well. In such case, manual detection would be time-consuming and the automatic detection method would be required.

For tracking the nuclei in time lapse images, we can apply the detection method to each time frame separately and then link the detected nuclei between frames. In this case, some false negatives and false positives would be separately produced for each frame, and they might disrupt the link step, resulting in increase of tracking errors. On the other hand, in the proposed method, the result of the automatic detection could be corrected manually, resulting in decrease of tracking errors.

The proposed tracking method is a simplistic approach. Combination with existing excellent tracking methods will likely improve tracking performance of the proposed method. Cell division and cell death did not occur in our data, but they are fundamental problems in the analysis of embryonic development. It may be important to improve our method if it is to be applied to these problems so that the method handles such phenomena appropriately.

## Methods

### Strains and cultures

*C*. *elegans* strains JN2100 and JN2101 were used in this study. Animals were raised on nematode growth medium at 20°C. *E*. *coli* strain OP50 was used as a food source.

#### JN2100

The genotype of the strain JN2100 is *Is[H20p*::*NLS4*::*mCherry]*. In this strain, the nuclei of all neurons are marked by the red fluorescent protein mCherry [42]. The *H20* promoter (*H20p*) is a 2479 bp DNA fragment derived from an upstream region of the *rimb-1* gene of *C*. *elegans* and drives gene expression in a pan-neuronal manner. mCherry was fused with four tandem repeats of nuclear localization signal (NLS) peptides and localized to the nuclei. The strain was generated by injection of an expression vector containing *H20p*::*NLS4*::*mCherry* to the Bristol N2 strain (wild type), integration of the transgene into the genome by UV irradiation, and outcrossing with N2 three times.

#### JN2101

The genotype of the strain JN2101 is *Is[H20p*::*NLS4*::*mCherry]; Ex [tax-4p*::*nls-YC2*.*60*, *lin-44p*::*GFP]*. In this strain, a genetically encoded calcium indicator (GECI) is expressed in some sensory neurons and reports the activity of these neurons. The *tax-4* promoter (*tax-4p*) is a 3132 bp DNA fragment derived from the upstream region of the *tax-4* gene of *C*. *elegans* and drives gene expression in a subset of sensory neurons including the ASER gustatory neuron [43]. *Yellow-Cameleon 2*.*60* (*YC2*.*60*) is a type of GECI and reports the Ca^2+^ concentration, which changes with neuronal activity, as the ratio of the fluorescence intensity of yellow fluorescent protein (YFP) to that of cyan fluorescent protein (CFP) [44]. YC2.60 was fused with an NLS peptide and localized mainly to the nuclei. The strain was generated by injection of an expression vector containing *tax-4p*::*nls-YC2*.*60* with a transformation marker (*lin-44p*::*GFP*, expressed in tail hypodermis cells) to JN2100.

### Datasets

We used three datasets in this study. Data1 and 2 contain ~200 neuronal nuclei, and Data3 contains 9 nuclei. The positions of the centers of the nuclei were manually corrected by experimental specialists using the proposed GUI.

#### Data 1

A set of static 3D single-channel images of strain JN2100 was used to test the nucleus detection performance of the proposed method. Day 1 adult animals were mounted on a 2% agar pad and paralyzed by sodium azide. The fluorescence of mCherry was observed using laser scanning confocal microscopy (Leica SP5 with 63× water immersion lens and 2× zoom). The sizes of the images along the *x*_1_ and *x*_2_ axes were 512 and 256 voxels, respectively, and the size along the *x*_3_ axis varied from 142 to 175 voxels depending on the diameter of the animal. The sizes of a voxel along the *x*_1_, *x*_2_, and *x*_3_ axes were 0.240, 0.240, and 0.252 μm, respectively.

#### Data 2

A time series of 3D multichannel images of strain JN2101 was used to test the tracking performance of the proposed method. A day 1 adult animal was introduced and held in a microfluidic device called olfactory chip [45]. The animal and its head neurons moved to some extent in the device because the animal was not paralyzed. The animal was stimulated from 50 to 100 s after initiation of each experiment with a down-step concentration of sodium chloride [39], and the fluorescence in the CFP, YFP, and mCherry channels was observed simultaneously using customized spinning disk confocal microscopy. The sizes of the image along the *x*_1_, *x*_2_, and *x*_3_ axes were 512, 256, and 20 voxels, respectively. The sizes of a voxel along the *x*_1_, *x*_2_, and *x*_3_ axes were 0.33, 0.33, and 1.40 μm, respectively. The volumetric frame rate was 4.75 per second (95 planar frame rate), and 591 3D frames were recorded (about 124 s).

#### Data 3

A time series of 2D multichannel images of strain JN2101 was used to test the detection and the tracking performance of the proposed method for 2D time lapse images. The experimental conditions for sample preparation and stimulation are same as Data 2 except stimulation period (51 to 151 frames after initiation of the experiment). The fluorescence in the CFP and YFP channels was observed simultaneously using DMI6000B inverted microscope with HCX PL APO 63x (NA 1.40) objective lens (Leica), a dual-view FRET imaging system DV2 (Photometrics) and an ImageEM EM-CCD camera (Hamamatsu Photonics) [46]. The sizes of the image along the *x*_1_ and *x*_2_ axes were 512 and 256, respectively. The sizes of a voxel along the *x*_1_ and *x*_2_ axes were 0.254 and 0.254 μm, respectively. The frame rate was 1.83 per second, and 241 2D frames were recorded (about 131 s).

### Outline of the proposed method

The blobs of the nuclei were detected by the conventional method (Steps 1 & 2). Under-segmented blobs were detected and split in Step 3. The precise positions and sizes of the nuclei were obtained in Step 4. The names and parameter values of the filters used in the proposed method are shown in S1 Table.

#### Step 1: Preprocessing

Parallel displacement along the *x*_1_ and *x*_2_ axes between images with different *x*_3_ or time axes was corrected on the basis of cross-correlation using the dftregistration function [47] implemented in MATLAB. If the data contained multiple channels or multiple time points, the images in one channel or one time point were extracted for nucleus detection. Next, the images were processed by a denoising filter (“Median 3D…” for Data 1), background subtraction (“Subtract Background…” for Data 1), and Gaussian blurring (“Gaussian Blur 3D…” for Data 1) using appropriate methods implemented in Fiji.

#### Step 2: Segmentation

Obvious background voxels in the images were removed by thresholding using an appropriate method implemented in Fiji. Local intensity peaks of the 3D image were detected using the 3D maximum filter implemented in MATLAB. If there were two or more peaks within the radius of the 3D maximum filter, only the brightest one was regarded as a local peak, and the others were discarded. The local peaks were used as seeds for 3D seeded grayscale watershed segmentation implemented as “Marker-controlled Watershed” in the MorphoLibJ plugin in Fiji. Too-small objects were regarded as background noise and removed. Background voxels in each segmented subimage were removed by thresholding [48] implemented in MATLAB.

#### Step 3: Clump splitting based on negative curvature

The curvature of the iso-intensity surface was calculated at each voxel (S2 Text) [28]. A segmented subimage was marked as under-segmented if the number of voxels having negative curvature in the subimage exceeded a threshold. If the voxels of negative curvature were not connected to the border of the subimage, they were regarded as noise and not counted. Voxels of negative curvature near the border of the subimage were regarded as part of a correct segmentation and not counted. If the subimages were marked as under-segmented, the voxels of negative curvature were removed, and the subimage was distance-transformed and segmented by the 3D seeded watershed algorithm.

#### Step 4: Least squares fitting with a Gaussian mixture

The positions, shapes, and intensities of detected nuclei were obtained by fitting with a Gaussian mixture. The least squares fitting procedure is described in detail in S1 Text and S4 Fig. The number of Gaussian distributions and the initial values of the centers of the distributions were derived from the results of clump splitting (previous step). The initial values of the covariance matrices of the distributions were fixed at a predefined default value. If the distance between the centers of two distributions was too small after fitting, either of the two distributions was removed, and the fitting procedure was repeated. After convergence, the scaling factors of the Gaussian distributions were estimated in other channels to obtain the intensities of the nuclei in these channels.

If the data contained multiple time points, the fitting procedure enabled tracking of the nuclei. The Gaussian mixture at the current time point was used as an initial value for the Gaussian mixture at the next time point, and the fitting procedure was executed without changing the eigenvalues of the covariance matrices (i.e., the size of the nuclei) [49]. This step was repeated for all the time points.

The proposed method was implemented as C, Java, and Matlab, then integrated as Matlab codes. In this study, the proposed method was tested on a desktop PC (3.2GHz 6-core Intel Core i7-3930K with 16GB memory, Windows 7 64bit). In order to confirm the portability of our method to Linux and cluster environment, the proposed method was tested on Shirokane3 supercomputing system (Human Genome Center, the University of Tokyo).

### Performance comparison

The performance of proposed method for cell detection was compared with five state-of-the-art methods: Ilastik, FARSight, RPHC, 3D watershed plugin in ImageJ, and CellSegmentation3D. Ilastik is machine learning-based method and required a training data that was created manually. The parameters of RPHC was the same as the literature [1]. The parameters of the other methods were optimized based on F-measure and accuracy. The parallel displacements of the raw 3D images of 12 animals in Data 1 was corrected, and the methods were applied to the images. Because FARSight crashed during processing, its command line version (segment_nuclei.exe) was used [50]. The input images for FARSight and CellSegmentation3D were converted to 8-bit images because they could not operate with 32-bit grayscale images. For CellSegmentation3D, because it could not operate with our whole 3D image, the input images were divided and processed separately. The comparison was performed and the processing time was measured on the same PC as that used for the proposed method. All the methods other than CellSegmentation3D might be able to utilize multi-threading.

The centroids of the segmented regions obtained by each program were used as the representative points of the objects. For the proposed method, the means of the fitted Gaussian distributions (*μ*_*k*_) were used as the representative points. The Euclid distances of the representative points and manually pointed Ground Truth were obtained. If a representative point was nearest-neighbor of a point of Ground Truth and vice versa, the object was regarded as a True Positive. If only the former condition was met, the Ground Truth was regarded as a False Negative. If only the latter condition was met, the object was regarded as a False Positive.

We obtained the indices of the performance [12,34,50] as follows:

$$True\;positive\;rate=\frac{TP}{GT},$$

$$False\;positive\;rate=\frac{FP}{GT},$$

$$False\;negative\;rate=\frac{FN}{GT},$$

$$F-measure=\frac{2\times TP}{2\times TP+FN+FP},$$

$$Accuracy=\frac{TP}{TP+FN+FP},$$

where

GT=TP+FN.

GT, TP, FP and FN mean Ground Truth, True Positive, False Positive and False Negative, respectively.

## Supporting Information

S1 Fig Shape and spatial distribution of nuclei obtained by the proposed method. (**A–C**) Radius of the nuclei in Data 1. The half-radii of fitted ellipsoids are obtained as the square roots of the eigenvalues of the covariance matrices of the fitted Gaussian distributions. (**D**) Shape of the nuclei measured as a ratio of maximum and minimum lengths of axes of the ellipsoids. (**E**) Distances to nearest neighbors of nuclei obtained as minimum distance between centers of the fitted Gaussian distributions. (**F**) Fluorescence intensities of nuclei obtained as the scaling parameters *π*_*k*_ of the fitted Gaussian distributions.
(EPS)

S2 Fig Screenshot of RoiEdit3D. (EPS)

S3 Fig A tracking result for 2-dimensional time series images. **(A)** The image at the first time point of Data 3 (2-dimsnional time series images) is shown. For presentation, only the CFP channel of the calcium indicator Yellow Cameleon is shown in light blue. ROI of ASER is shown as a yellow ellipse. Other ROIs appear as blue ellipses. **(B)** Time course of ASER response obtained by the calcium indicator. Gray area indicates the stimulation period.
(EPS)

S4 Fig Verification of proposed alternate optimization procedure. Time courses indicate the progress of the optimization procedures for the 3D images of 12 animals in Data 1. Table shows fitness scores of procedures for each animal and the number of trials having scores within 110% of the best score.
(EPS)

S1 Table Names and parameter values of filters used in the proposed method. (DOCX)

S1 Text Procedure for least squares fitting with a Gaussian mixture. (DOCX)

S2 Text Procedure for calculation of principal curvatures of iso-intensity surfaces. (DOCX)
