# Supplementary material for: Accurate Automatic Detection of Densely Distributed Cell Nuclei in 3D Space
Source: PLoS Comput Biol. 2016 Jun 6;12(6):e1004970. doi: 10.1371/journal.pcbi.1004970 (PMC4894571; doi:10.1371/journal.pcbi.1004970)
Supplement: S1 Table — (DOCX) [file pcbi.1004970.s005.docx]

**S1 Table: Names and parameter values of filters used in the proposed method.**

| **Steps** | **Process** | **Contents** | **For Data 1** | **For Data 2** |
| --- | --- | --- | --- | --- |
| Step 1 | Denoising | Filter name | Median 3D… | Median |
|  |  | Radius | x=2 y=2 z=2 | radius=2 |
|  | Background subtraction | Filter name | Subtract Background… | Subtract Background… |
|  |  | Radius | 50 | 50 |
|  | Gaussian blurring | Filter name | Gaussian Blur 3D… | Gaussian Blur… |
|  |  | Radius | x=2 y=2 z=2 | radius=2 |
| Step 2 | Thresholding | Method name | Mean | Triangle |
|  | 3D maximum filter | Radius | 5 | 8 |
|  | Removing too-small objects | Minimum object size | 64 | 1000 |
| Step 3 | Counting numbers of voxels of negative curvature | Threshold | 50 | 100 |
|  |  | Distance to borders | 5 | 7 |
| Step 4 | Least squares fitting of Gaussian mixture | Default value of covariance matrix | diag(10,10,10) | diag(9,9,1.5) |
|  | Removing over-segmentation | Distance | 1.9 [μm] | 1.5 [μm] |

Filter names are plugin names in Fiji. Units of parameter values are voxels unless otherwise noted.
