# Supplementary material for: Accurate Automatic Detection of Densely Distributed Cell Nuclei in 3D Space
Source: PLoS Comput Biol. 2016 Jun 6;12(6):e1004970. doi: 10.1371/journal.pcbi.1004970 (PMC4894571; doi:10.1371/journal.pcbi.1004970)
Supplement: S1 Text — (DOCX) [file pcbi.1004970.s006.docx]

**S1 Text: Procedure for least squares fitting with a Gaussian mixture**

The nuclei in our dataset have roughly ellipsoidal forms, and the intensity is higher near the centers of the nuclei. We can approximate the intensity of the nuclei by a mixture of trivariate Gaussian distributions. Gaussian mixture models have been applied to images with many particles using conventional optimization procedures [1–3]. Standard optimization procedures such as the expectation–maximization algorithm consider a Gaussian mixture to be a probabilistic distribution and maximize the likelihood of obtaining the data as a frequency distribution. Therefore, in previous applications to images, the voxel intensities corresponded to the frequency of data that came from a Gaussian mixture probability, and only a small noise was tolerated at positions distant from the peak. However, when the density distribution of a cell (or nucleus) is approximated by a Gaussian function, a different model is more appropriate. Namely, we consider a Gaussian function as a mean of the intensity distribution for each voxel and minimize the sum of the squares of the residuals from this mean as follows.

The intensity $f_{k}$ of the $k$-th Gaussian distribution$g_{k}$ at voxel position $x\in\mathbb{R}^{3}$ is

$$f_{k}\left( x \right)=\pi_{k}g_{k}\left( x | \mu_{k},\Sigma_{k} \right)$$

$$=\pi_{k}\exp\left( -\frac{1}{2}\left( x-\mu_{k} \right)^{\text{T}}\Sigma_{k}^{-1}\left( x-\mu_{k} \right) \right),$$

where $\mu_{k}$ and $\Sigma_{k}$ are the mean vector and covariance matrix of $g_{k}$, respectively, and $\pi_{k}$ is an intensity scaling factor. When an image includes $K$ pieces of nuclei, we use a mixture of $K$ pieces of Gaussian distribution,

$$f\left( x \right)=\sum_{k \in K} f_{k}\left( x \right),$$

and the intensity of the image, $y\left( x \right)$, can be approximated as

$y\left( x \right)\sim N\left( f\left( x \right),\sigma^{2} \right)$,

where $N(\mu,\sigma^{2})$ is a normal distribution with a mean $\mu$ and variance $\sigma^{2}$. The likelihood for the image is

$$P=\prod_{x} P\left( y\left( x \right) \right)$$

$$=\prod_{x} \frac{1}{\sqrt{2\pi\sigma^{2}}}\exp\left( -\frac{1}{2\sigma^{2}}\left( y\left( x \right)-f\left( x \right) \right)^{2} \right),$$

$$\log\left( P \right)=\sum_{x} \left( -\frac{1}{2\sigma^{2}}\left( y\left( x \right)-f\left( x \right) \right)^{2}-\log\left( \sqrt{2\pi\sigma^{2}} \right) \right),$$

$$L\left( \pi,\theta\right)=\sum_{x} r\left( x | \theta\right)$$

$$=\sum_{x} \left( y\left( x \right)-f\left( x \right) \right)^{2}$$

$$=\sum_{x} \left( y\left( x \right)- \sum_{k\in K} \pi_{k}g\left( x | \theta_{k} \right) \right)^{2},$$

and we search for the parameters $\pi_{k}$ and $\theta_{k}=\left( \mu_{k},\Sigma_{k} \right)$ that minimize the sum of the squared residuals $L$. Although this is a typical nonlinear least square problem, searching for the parameters using a general solver is difficult because the numbers of voxels and parameters are very large. Thus, we propose an iterative alternate optimization procedure. An iteration of the procedure consists of the four steps

update $\pi_{k}$ -> update $\mu_{k}$ -> update $\pi_{k}$ -> update $\Sigma_{k}$,

using equations (1)–(3) below, and iteration is performed until $L$ converges.

The proposed alternate optimization procedure was compared with general gradient-based optimization procedures. The functions fmincon and fminunc in MATLAB 2012b were used for the general gradient-based solver. The solver minimized $L$ using custom-written C-Mex code that calculates the gradient and/or Hessian of $L$. The proposed alternate optimization procedure achieved good scores (within 110% of the best scores) in all 12 trials (S3 Fig). In contrast, the general gradient-based optimization procedure often failed to reach good scores.

The following sections describe the derivation of equations (1)–(3).

$\mathbf{For updating}\boldsymbol{\pi:}$

The partial derivative of $L$ by $\pi_{k}$ is $0$ at the minimum of $L$, so we have

$$\frac{\partial L}{\partial\pi_{k}}=\sum_{x} \left( 2g\left( x| \theta_{k} \right)\left( y\left( x \right)-\sum_{\kappa\in K} \pi_{\kappa}g\left( x | \theta_{\kappa} \right) \right) \right)$$

$$=0,$$

$$\sum_{x} \left( g\left( x | \theta_{k} \right)y(x) \right)=\sum_{\kappa\in K} \pi_{\kappa}\sum_{x} \left( g\left( x | \theta_{k} \right)g\left( x | \theta_{\kappa} \right) \right). -------- (1)$$

If we assume $\theta_{k}$ are constants, the above is a series of linear equations for $\pi_{k}$, and we can obtain $\pi_{k}$ directly by linear algebra.

**For updating** $\boldsymbol{\mu:}$

We apply the Gauss–Newton method to minimize $\sum_{x} r\left( x|\mu\right)^{2}$, and we obtain the update equation for $\mu$ as

$$\mu^{(l+1)}=\mu^{(l)}-\left( J_{\mu}^{\text{T}}J_{\mu} \right)^{-1}J_{\mu}^{\text{T}}r\left( x|\mu^{\left( l \right)} \right),$$

where $l$ is the number of iteration steps in the Gauss–Newton method, and $J_{\mu}$ is the Jacobian matrix of $r(x|\mu)$,

$$J_{\mu}=\frac{\partial r(x|\mu)}{\partial\mu}.$$

For the $k$-th Gaussian distribution, we have

$$\mu_{k}^{\left( l+1 \right)}=\mu_{k}^{\left( l \right)}-\sum_{\kappa} \left\{ \left( J_{\mu}^{\text{T}}J_{\mu} \right)^{-1} \right\}_{k,\kappa}\left\{ J_{\mu}^{\text{T}}r\left( x | \mu^{\left( l \right)} \right) \right\}_{\kappa},$$

where

$$\left( J_{\mu} \right)_{k}=\frac{\partial r\left( x|\mu\right)}{\partial\mu_{k}}$$

$$=\frac{\partial}{\partial\mu_{k}}\left\{ y-\sum_{\kappa} \pi_{\kappa}g_{\kappa} \right\}$$

$$=-\pi_{k}g_{k}\Sigma_{k}^{-1}\left( x-\mu_{k} \right).$$

Then

$$\left\{ J_{\mu}^{T}r\left( x|\mu\right) \right\}_{\kappa}=\sum_{x} \left( J_{\mu} \right)_{\kappa}r\left( x|\mu\right)$$

$$=\sum_{x} -\pi_{\kappa}g_{\kappa}\Sigma_{\kappa}^{-1}\left( x-\mu_{\kappa} \right)r\left( x|\mu\right)$$

$$=-\pi_{\kappa}\Sigma_{\kappa}^{-1}\sum_{x} r\left( x | \mu\right)g_{\kappa}\left( x-\mu_{\kappa} \right),$$

$$\left( J_{\mu}^{\text{T}}J_{\mu} \right)_{k,\kappa}=\sum_{x} \left( J_{\mu}^{\text{T}} \right)_{k}\left( J_{\mu} \right)_{\kappa}$$

$$=\sum_{x} \left\{ \pi_{k}g_{k}\Sigma_{k}^{-1}\left( x-\mu_{k} \right) \right\}\left\{ \pi_{\kappa}g_{\kappa}\Sigma_{\kappa}^{-1}\left( x-\mu_{\kappa} \right) \right\}^{\text{T}}$$

$$=\pi_{k}\pi_{\kappa}\sum_{x} g_{k}g_{\kappa}\Sigma_{k}^{-1}\left( x-\mu_{k} \right)\left( x-\mu_{\kappa} \right)^{\text{T}}\Sigma_{\kappa}^{-1}.$$

Here, the nondiagonal elements of the matrix $J_{\mu}^{\text{T}}J_{\mu}$, namely, $\left( J_{\mu}^{\text{T}}J_{\mu} \right)_{k,\kappa} \left( k\neq\kappa\right)$, are smaller than the diagonal elements, $\left( J_{\mu}^{\text{T}}J_{\mu} \right)_{k,k}$ $(k=\kappa)$, and can be ignored in the following approximation:

$$\left( J_{\mu}^{\text{T}}J_{\mu} \right)_{k,\kappa}= \delta_{k,\kappa}\pi_{k}\pi_{\kappa}\sum_{x} g_{k}g_{\kappa}\Sigma_{k}^{-1}\left( x-\mu_{k} \right)\left( x-\mu_{\kappa} \right)^{\text{T}}\Sigma_{\kappa}^{-1}$$

$$=\delta_{k,\kappa}\pi_{k}^{2}\Sigma_{k}^{-1}\sum_{x} g_{k}^{2}\left( x-\mu_{k} \right)\left( x-\mu_{k} \right)^{T}\Sigma_{k}^{-1}$$

$$=\delta_{k,\kappa}\pi_{k}^{2}\Sigma_{k}^{-1}\sum_{x} \exp\left\{ -\left( x-\mu\right)^{T}\Sigma_{k}^{-1}\left( x-\mu\right) \right\}\left( x-\mu_{k} \right)\left( x-\mu_{k} \right)^{T}\Sigma_{k}^{-1}$$

$$=\delta_{k,\kappa}\pi_{k}^{2}\Sigma_{k}^{-1}\sum_{x} \exp\left\{ -{\frac{1}{2}\left( x-\mu\right)}^{T}\left( \frac{\Sigma_{k}}{2} \right)^{-1}\left( x-\mu\right) \right\}\left( x-\mu_{k} \right)\left( x-\mu_{k} \right)^{T}\Sigma_{k}^{-1}$$

$$=\delta_{k,\kappa}\pi_{k}^{2}\Sigma_{k}^{-1}\sum_{x} \left( 2\pi\right)^{\frac{3}{2}}\left| \frac{\Sigma_{k}}{2} \right|^{\frac{1}{2}}N\left( x|\mu_{k},\frac{\Sigma_{k}}{2} \right)\left( x-\mu_{k} \right)\left( x-\mu_{k} \right)^{T}\Sigma_{k}^{-1}$$

$$=\delta_{k,\kappa}\pi_{k}^{2}\Sigma_{k}^{-1}\left( 2\pi\right)^{\frac{3}{2}}\left| \frac{\Sigma_{k}}{2} \right|^{\frac{1}{2}}\frac{\Sigma_{k}}{2}\Sigma_{k}^{-1}$$

$$=\delta_{k,\kappa}\frac{\pi_{k}^{2}}{2^{\frac{3}{2}}}\left( 2\pi\right)^{\frac{3}{2}}\left| \Sigma_{k} \right|^{\frac{1}{2}}\Sigma_{k}^{-1}.$$

Then we obtain the update equation for $\mu$,

$$\mu_{k}^{\left( l+1 \right)}=\mu_{k}^{\left( l \right)}-\sum_{\kappa} \left\{ \left( J_{\mu}^{\text{T}}J_{\mu} \right)^{-1} \right\}_{k,\kappa}\left\{ J_{\mu}^{\text{T}}r\left( x | \mu^{\left( l \right)} \right) \right\}_{\kappa}$$

$$=\mu_{k}^{\left( l \right)}-\sum_{\kappa} \delta_{k,\kappa}\frac{-\pi_{\kappa}\Sigma_{\kappa}^{-1}\sum_{x} r\left( x | \mu\right)g_{\kappa}\left( x-\mu_{\kappa} \right)}{\frac{\pi_{k}^{2}}{2^{\frac{3}{2}}}\left( 2\pi\right)^{\frac{3}{2}}\left| \Sigma_{k} \right|^{\frac{1}{2}}\Sigma_{k}^{-1}}$$

$$=\mu_{k}^{\left( l \right)}+\frac{\sum_{x} r\left( x | \mu\right)g_{k}\left( x-\mu_{k} \right)}{\frac{\pi_{k}}{2^{\frac{3}{2}}}\left( 2\pi\right)^{\frac{3}{2}}\left| \Sigma_{k} \right|^{\frac{1}{2}}}.----------(2)$$

**For updating** $\boldsymbol{\Sigma:}$

We apply Gauss-Newton method to minimize $\sum_{x} r\left( x|\Sigma\right)^{2}$ and we obtain update equation of $\Sigma$ as

$$\Sigma^{\left( l+1 \right)}=\Sigma^{\left( l \right)}-\left( J_{\Sigma}^{\text{T}}J_{\Sigma} \right)^{-1}J_{\Sigma}^{T}r\left( x | \Sigma^{\left( l \right)} \right)$$

or

$$J_{\Sigma}^{T}r\left( x | \Sigma^{\left( l \right)} \right)=-\left( J_{\Sigma}^{\text{T}}J_{\Sigma} \right)\left( \Sigma^{\left( l+1 \right)}-\Sigma^{\left( l \right)} \right)$$

$$=-\left( J_{\Sigma}^{\text{T}}J_{\Sigma} \right)\Delta\Sigma,$$

where $J_{\Sigma}$ is the Jacobian matrix of $r\left( x|\Sigma\right)$, and $\Delta\Sigma=\left( \Sigma^{\left( l+1 \right)}-\Sigma^{\left( l \right)} \right)$. For the $k$-th Gaussian distribution, we have

$$\left\{ J_{\Sigma}^{T}r\left( x | \Sigma^{\left( l \right)} \right) \right\}_{k,p,q}=-\sum_{\kappa} \sum_{r} \sum_{s} \left( J_{\Sigma}^{\text{T}}J_{\Sigma} \right)_{k,p,q,\kappa,r,s}\Delta\Sigma_{\kappa,r,s},$$

where $\Sigma_{k,p,q}$ is the $\left( p,q \right)$-th element of $\Sigma_{k}$, and

$$\left( J_{\Sigma} \right)_{k}=\frac{\partial r\left( x | \Sigma\right)}{\partial\Sigma_{k}}$$

$$=\frac{\partial}{\partial\Sigma_{k}}\left\{ y-\sum_{\kappa} \pi_{\kappa}g_{\kappa} \right\}$$

$$=-\frac{1}{2}\pi_{k}g_{k}\Sigma_{k}^{-1}\left( x-\mu_{k} \right)\left( x-\mu_{k} \right)^{T}\Sigma_{k}^{-1}.$$

Then,

$$\left\{ J_{\Sigma}^{T}r\left( x|\Sigma\right) \right\}_{k,p,q}=\left\{ \sum_{x} \left( J_{\Sigma} \right)_{k}r\left( x|\Sigma\right) \right\}_{p,q}$$

$$=\left\{ \sum_{x} \left\{ -\frac{1}{2}\pi_{k}g_{k}\Sigma_{k}^{-1}\left( x-\mu_{k} \right)\left( x-\mu_{k} \right)^{T}\Sigma_{k}^{-1} \right\}r\left( x|\Sigma\right) \right\}_{p,q}$$

$$=\left\{ -{\frac{1}{2}\pi}_{k}\sum_{x} r\left( x | \Sigma\right)g_{k}\Sigma_{k}^{-1}\left( x-\mu_{k} \right)\left( x-\mu_{k} \right)^{T}\Sigma_{k}^{-1} \right\}_{p,q},$$

$$\left( J_{\Sigma}^{\text{T}}J_{\Sigma} \right)_{k,p,q,\kappa,r,s}=\sum_{x} \left\{ -\frac{1}{2}\pi_{k}g_{k}\Sigma_{k}^{-1}\left( x-\mu_{k} \right)\left( x-\mu_{k} \right)^{T}\Sigma_{k}^{-1} \right\}_{p,q}\times\left\{ -{\frac{1}{2}\pi}_{\kappa}g_{\kappa}\Sigma_{\kappa}^{-1}\left( x-\mu_{\kappa} \right)\left( x-\mu_{\kappa} \right)^{T}\Sigma_{\kappa}^{-1} \right\}_{r,s}$$

$$=\frac{1}{4}\pi_{k}\pi_{\kappa}\sum_{x} g_{k}{g_{\kappa}\left\{ \Sigma_{k}^{-1}\left( x-\mu_{k} \right)\left( x-\mu_{k} \right)^{T}\Sigma_{k}^{-1} \right\}}_{p,q}\times\left\{ \Sigma_{\kappa}^{-1}\left( x-\mu_{\kappa} \right)\left( x-\mu_{k} \right)^{T}\Sigma_{\kappa}^{-1} \right\}_{r,s}.$$

Here, the nondiagonal elements of the matrix $J_{\Sigma}^{\text{T}}J_{\Sigma}$, namely, $\left( J_{\Sigma}^{\text{T}}J_{\Sigma} \right)_{k,\kappa} \left( k\neq\kappa\right)$, are smaller than the diagonal elements, $\left( J_{\Sigma}^{\text{T}}J_{\Sigma} \right)_{k,k}$ $(k=\kappa)$, and can be ignored in the following approximation:

$$\left( J_{\Sigma}^{\text{T}}J_{\Sigma} \right)_{k,p,q,\kappa,r,s}= \delta_{k,\kappa}\frac{1}{4}\pi_{k}\pi_{\kappa}\sum_{x} g_{k}{g_{\kappa}\left\{ \Sigma_{k}^{-1}\left( x-\mu_{k} \right)\left( x-\mu_{k} \right)^{T}\Sigma_{k}^{-1} \right\}}_{p,q}\times\left\{ \Sigma_{\kappa}^{-1}\left( x-\mu_{\kappa} \right)\left( x-\mu_{k} \right)^{T}\Sigma_{\kappa}^{-1} \right\}_{r,s}$$

$$=\delta_{k,\kappa}\frac{1}{4}\pi_{k}^{2}\sum_{x} g_{k}^{2}\left\{ \Sigma_{k}^{-1}\left( x-\mu_{k} \right)\left( x-\mu_{k} \right)^{T}\Sigma_{k}^{-1} \right\}_{p,q}\times\left\{ \Sigma_{k}^{-1}\left( x-\mu_{k} \right)\left( x-\mu_{k} \right)^{T}\Sigma_{k}^{-1} \right\}_{r,s}$$

$$=\delta_{k,\kappa}\frac{1}{4}\pi_{k}^{2}\sum_{x} \left( 2\pi\right)^{\frac{3}{2}}\left| \frac{\Sigma_{k}}{2} \right|^{\frac{1}{2}}N\left( x|\mu_{k},\frac{\Sigma_{k}}{2} \right)\left\{ \Sigma_{k}^{-1}\left( x-\mu_{k} \right)\left( x-\mu_{k} \right)^{T}\Sigma_{k}^{-1} \right\}_{p,q}\times\left\{ \Sigma_{k}^{-1}\left( x-\mu_{k} \right)\left( x-\mu_{k} \right)^{T}\Sigma_{k}^{-1} \right\}_{r,s}.$$

Let $u=x-\mu$, and we have

$$\int_{x} \left[ N\left( x | \mu,\frac{\Sigma}{2} \right)\left\{ \Sigma^{-1}\left( x-\mu\right)\left( x-\mu\right)^{T}\Sigma^{-1} \right\}_{p,q}\left\{ \Sigma^{-1}\left( x-\mu\right)\left( x-\mu\right)^{T}\Sigma^{-1} \right\}_{r,s} \right]$$

$$=\int_{x} \left[ N\left( x | \mu,\frac{\Sigma}{2} \right)\left\{ \Sigma^{-1}uu^{T}\Sigma^{-1} \right\}_{p,q}\left\{ \Sigma^{-1}uu^{T}\Sigma^{-1} \right\}_{r,s} \right]$$

$$=\int_{x} \left[ N\left( x | \mu,\frac{\Sigma}{2} \right)\left\{ \Sigma^{-1}u\left( \Sigma^{-1}u \right)^{T} \right\}_{p,q}\left\{ \Sigma^{-1}u\left( \Sigma^{-1}u \right)^{T} \right\}_{r,s} \right]$$

$$=\int_{x} \left[ N\left( x | \mu,\frac{\Sigma}{2} \right)\left\{ \left( \sum_{i} \left( \Sigma^{-1} \right)_{i,p}u_{i} \right)\left( \sum_{j} \left( \Sigma^{-1} \right)_{j,q}u_{j} \right) \right\}\left\{ \left( \sum_{k} \left( \Sigma^{-1} \right)_{k,r}u_{k} \right)\left( \sum_{l} \left( \Sigma^{-1} \right)_{l,s}u_{l} \right) \right\} \right]$$

$$=\sum_{i} \sum_{j} \sum_{k} \sum_{l} \left( \Sigma^{-1} \right)_{i,p}\left( \Sigma^{-1} \right)_{j,q}\left( \Sigma^{-1} \right)_{k,r}\left( \Sigma^{-1} \right)_{l,s}\int_{x} \left[ N\left( x | \mu,\frac{\Sigma}{2} \right)\left\{ u_{i}u_{j}u_{k}u_{l} \right\} \right]$$

$$=\sum_{i} \sum_{j} \sum_{k} \sum_{l} \left( \Sigma^{-1} \right)_{i,p}\left( \Sigma^{-1} \right)_{j,q}\left( \Sigma^{-1} \right)_{k,r}\left( \Sigma^{-1} \right)_{l,s}\left\{ \frac{1}{4}\left( \Sigma_{i,j}\Sigma_{k,l}+\Sigma_{i,k}\Sigma_{j,l}+\Sigma_{i,l}\Sigma_{j,k} \right) \right\}$$

$$=\frac{1}{4}\sum_{i} \sum_{j} \left( \Sigma^{-1} \right)_{i,p}\left( \Sigma^{-1} \right)_{j,q}\Sigma_{i,j}\sum_{k} \sum_{l} \left( \Sigma^{-1} \right)_{k,r}\left( \Sigma^{-1} \right)_{l,s}\Sigma_{k,l}+\frac{1}{4}\sum_{i} \sum_{k} \left( \Sigma^{-1} \right)_{i,p}\left( \Sigma^{-1} \right)_{k,r}\Sigma_{i,k}\sum_{j} \sum_{l} \left( \Sigma^{-1} \right)_{j,q}\left( \Sigma^{-1} \right)_{l,s}\Sigma_{j,l}+\frac{1}{4}\sum_{i} \sum_{l} \left( \Sigma^{-1} \right)_{i,p}\left( \Sigma^{-1} \right)_{l,s}\Sigma_{i,l}\sum_{j} \sum_{k} \left( \Sigma^{-1} \right)_{j,q}\left( \Sigma^{-1} \right)_{k,r}\Sigma_{j,k}$$

$$=\frac{1}{4}\left\{ \left( \Sigma^{-1} \right)_{p,q}\left( \Sigma^{-1} \right)_{r,s}+\left( \Sigma^{-1} \right)_{p,r}\left( \Sigma^{-1} \right)_{q,s}+\left( \Sigma^{-1} \right)_{p,s}\left( \Sigma^{-1} \right)_{q,r} \right\}.$$

Then, we have

$$\left( J_{\Sigma}^{\text{T}}J_{\Sigma} \right)_{k,p,q,\kappa,r,s}=\delta_{k,\kappa}\frac{1}{4}\pi_{k}^{2}\left( 2\pi\right)^{\frac{3}{2}}\left| \frac{\Sigma_{k}}{2} \right|^{\frac{1}{2}}\frac{1}{4}\left\{ \left( \Sigma_{k}^{-1} \right)_{p,q}\left( \Sigma_{k}^{-1} \right)_{r,s}+\left( \Sigma_{k}^{-1} \right)_{p,r}\left( \Sigma_{k}^{-1} \right)_{q,s}+\left( \Sigma_{k}^{-1} \right)_{p,s}\left( \Sigma_{k}^{-1} \right)_{q,r} \right\},$$

and

$$-\sum_{\kappa} \sum_{r} \sum_{s} \left( J_{\Sigma}^{\text{T}}J_{\Sigma} \right)_{k,p,q,\kappa,r,s}\Delta\Sigma_{\kappa,r,s}$$

$$=-\sum_{\kappa} \sum_{r} \sum_{s} \delta_{k,\kappa}\frac{1}{4}\pi_{k}^{2}\left( 2\pi\right)^{\frac{3}{2}}\left| \frac{\Sigma_{k}}{2} \right|^{\frac{1}{2}}\frac{1}{4}\left\{ \left( \Sigma_{k}^{-1} \right)_{p,q}\left( \Sigma_{k}^{-1} \right)_{r,s}+\left( \Sigma_{k}^{-1} \right)_{p,r}\left( \Sigma_{k}^{-1} \right)_{q,s}+\left( \Sigma_{k}^{-1} \right)_{p,s}\left( \Sigma_{k}^{-1} \right)_{q,r} \right\}\Delta\Sigma_{\kappa,r,s}$$

$$=-\sum_{r} \sum_{s} \frac{\pi_{k}^{2}}{2^{\frac{9}{2}}}\left( 2\pi\right)^{\frac{3}{2}}\left| \Sigma_{k} \right|^{\frac{1}{2}}\left\{ \left( \Sigma_{k}^{-1} \right)_{p,q}\left( \Sigma_{k}^{-1} \right)_{r,s}+\left( \Sigma_{k}^{-1} \right)_{p,r}\left( \Sigma_{k}^{-1} \right)_{q,s}+\left( \Sigma_{k}^{-1} \right)_{p,s}\left( \Sigma_{k}^{-1} \right)_{q,r} \right\}\Delta\Sigma_{k,r,s}$$

$$=-\frac{\pi_{k}^{2}}{2^{\frac{9}{2}}}\left( 2\pi\right)^{\frac{3}{2}}\left| \Sigma_{k} \right|^{\frac{1}{2}}\left\{ \sum_{r} \sum_{s} \left( \Sigma_{k}^{-1} \right)_{p,q}\left( \Sigma_{k}^{-1} \right)_{r,s}\Delta\Sigma_{k,r,s}+\sum_{r} \sum_{s} \left( \Sigma_{k}^{-1} \right)_{p,r}{\Delta\Sigma_{k,r,s}\left( \Sigma_{k}^{-1} \right)}_{q,s}+\sum_{r} \sum_{s} \left( \Sigma_{k}^{-1} \right)_{p,s}\Delta\Sigma_{k,r,s}\left( \Sigma_{k}^{-1} \right)_{q,r} \right\}$$

$$=-\frac{\pi_{k}^{2}}{2^{\frac{9}{2}}}\left( 2\pi\right)^{\frac{3}{2}}\left| \Sigma_{k} \right|^{\frac{1}{2}}\left\{ \left( \Sigma_{k}^{-1} \right)_{p,q}\sum_{r} \sum_{s} \left( \Sigma_{k}^{-1} \right)_{r,s}\Delta\Sigma_{k,r,s}+\left\{ \Sigma_{k}^{-1}\Delta\Sigma_{k}\Sigma_{k}^{-1} \right\}_{p,q}+\left\{ \Sigma_{k}^{-1}\Delta\Sigma_{k}\Sigma_{k}^{-1} \right\}_{p,q} \right\}$$

$$=-\frac{\pi_{k}^{2}}{2^{\frac{9}{2}}}\left( 2\pi\right)^{\frac{3}{2}}\left| \Sigma_{k} \right|^{\frac{1}{2}}\left\{ \left( \Sigma_{k}^{-1} \right)_{p,q}\mathrm{Tr}\left( \Sigma_{k}^{-1}\Delta\Sigma_{k} \right)+{2\left( \Sigma_{k}^{-1}\Delta\Sigma_{k}\Sigma_{k}^{-1} \right)}_{p,q} \right\}.$$

Applying these results to the update equation for $\Sigma$ for the $k$-th Gaussian distribution, we have

$$\left\{ -{\frac{1}{2}\pi}_{k}\sum_{x} r\left( x | \Sigma\right)g_{k}\Sigma_{k}^{-1}\left( x-\mu_{k} \right)\left( x-\mu_{k} \right)^{T}\Sigma_{k}^{-1} \right\}_{p,q}=\frac{\pi_{k}^{2}}{2^{\frac{9}{2}}}\left( 2\pi\right)^{\frac{3}{2}}\left| \Sigma_{k} \right|^{\frac{1}{2}}\left\{ \mathrm{Tr}\left( \Sigma_{k}^{-1}\Delta\Sigma_{k} \right)\Sigma_{k}^{-1}+{2\Sigma}_{k}^{-1}\Delta\Sigma_{k}\Sigma_{k}^{-1} \right\}_{p,q},$$

$$\frac{\sum_{x} r\left( x | \Sigma\right)g_{k}\left( x-\mu_{k} \right)\left( x-\mu_{k} \right)^{T}}{\frac{\pi_{k}}{2^{\frac{7}{2}}}\left( 2\pi\right)^{\frac{3}{2}}\left| \Sigma_{k} \right|^{\frac{1}{2}}}=\mathrm{Tr}\left( \Sigma_{k}^{-1}\Delta\Sigma_{k} \right)\Sigma_{k}+2\Delta\Sigma_{k}.$$

Here we use the vectorization operator $vec()$ and half-vectorization operator $vech()$ to expand the trace term,

$$\mathrm{Tr}\left( \Sigma_{k}^{-1}\Delta\Sigma_{k} \right)\Sigma_{k}=\mathrm{vec}\left( \Sigma_{k}^{-1} \right)^{T}\mathrm{vec}\left( \Delta\Sigma_{k} \right)\mathrm{vech}\left( \Sigma_{k} \right)$$

$$=\left\{ D_{m} \mathrm{vech}\left( \Sigma_{k}^{-1} \right) \right\}^{T}D_{m}\mathrm{vech}\left( \Delta\Sigma_{k} \right)\mathrm{vech}\left( \Sigma_{k} \right)$$

$$=vech\left( \Sigma_{k} \right)\mathrm{vech}\left( \Sigma_{k}^{-1} \right)^{T}D_{m}^{T}D_{m}\mathrm{vech}\left( \Delta\Sigma_{k} \right),$$

where the duplication matrix $D_{m}$ satisfies $\mathrm{vec}\left( A \right)=D_{m}\mathrm{vech}\left( A \right)$ for symmetric matrix $A$. Finally,

$$\mathrm{vech}\left( \frac{\sum_{x} r\left( x | \Sigma\right)g_{k}\left( x-\mu_{k} \right)\left( x-\mu_{k} \right)^{T}}{\frac{\pi_{k}}{2^{\frac{7}{2}}}\left( 2\pi\right)^{\frac{3}{2}}\left| \Sigma_{k} \right|^{\frac{1}{2}}} \right)=\mathrm{vech}\left( \Sigma_{k} \right)\mathrm{vech}\left( \Sigma_{k}^{-1} \right)^{T}D_{m}^{T}D_{m}\mathrm{vech}\left( \Delta\Sigma_{k} \right) +2\mathrm{vech}\left( \Delta\Sigma_{k} \right),$$

$$\mathrm{vech}\left( \Delta\Sigma_{k} \right)=\left\{ \mathrm{vech}\left( \Sigma_{k} \right)\mathrm{vech}\left( \Sigma_{k}^{-1} \right)^{T}D_{m}^{T}D_{m}+2I \right\}^{-1}\mathrm{vech}\left( \frac{\sum_{x} r\left( x | \Sigma\right)g_{k}\left( x-\mu_{k} \right)\left( x-\mu_{k} \right)^{T}}{\frac{\pi_{k}}{2^{\frac{7}{2}}}\left( 2\pi\right)^{\frac{3}{2}}\left| \Sigma_{k} \right|^{\frac{1}{2}}} \right),$$

$$\mathrm{vech}\left( \Sigma_{k}^{\left( l+1 \right)} \right)=\mathrm{vech}\left( \Sigma_{k}^{\left( l \right)} \right)+\left\{ \mathrm{vech}\left( \Sigma_{k}^{\left( l \right)} \right)\mathrm{vech}\left( \Sigma_{k}^{\left( l \right),-1} \right)^{T}D_{m}^{T}D_{m}+2I \right\}^{-1}\times vech\left( \frac{\sum_{x} r\left( x | \Sigma^{\left( l \right)} \right)g_{k}\left( x-\mu_{k} \right)\left( x-\mu_{k} \right)^{T}}{\frac{\pi_{k}}{2^{\frac{7}{2}}}\left( 2\pi\right)^{\frac{3}{2}}\left| \Sigma_{k}^{\left( l \right)} \right|^{\frac{1}{2}}} \right). -------(3)$$

We use this equation to update $\Sigma$.

If $\Sigma_{k}^{\left( l+1 \right)}$ was not positive-definite, the update was discarded; i.e., $\Sigma_{k}^{\left( l+1 \right)}= \Sigma_{k}^{\left( l \right)}$.

When the eigenvalue of $\Sigma_{k}$ should be fixed, the eigenvalue of $\Sigma_{k}^{\left( l+1 \right)}$ was replaced with the eigenvalue of $\Sigma_{k}^{\left( l \right)}$.

The whole procedure for least squares fitting with a Gaussian mixture was implemented as C language. The most time-consuming step in the algorithm is the calculation of intensity of Gaussian distributions. The calculation of intensity was restricted to a small neighborhood around the distribution, and parallelized for each distribution. The average processing time for the Gaussian fitting was 0.197 sec per step and 831.58 steps per animal for Data 1.

**References for S1 Text**

1. Pan K, Kokaram A, Hillebrand J, Ramaswami M. Gaussian mixtures for intensity modeling of spots in microscopy. 2010 IEEE International Symposium on Biomedical Imaging: From Nano to Macro. IEEE; 2010. pp. 121–124. doi:10.1109/ISBI.2010.5490398

2. Liang L, Shen H, De Camilli P, Duncan JS. Tracking clathrin coated pits with a multiple hypothesis based method. Lecture Notes in Computer Science (including subseries Lecture Notes in Artificial Intelligence and Lecture Notes in Bioinformatics). 2010. pp. 315–322. doi:10.1007/978-3-642-15745-5_39

3. Amat F, Lemon W, Mossing DP, McDole K, Wan Y, Branson K, et al. Fast, accurate reconstruction of cell lineages from large-scale fluorescence microscopy data. Nat Methods. 2014;11: 951–958. doi:10.1038/nmeth.3036
