# Supplementary material for: Accurate Automatic Detection of Densely Distributed Cell Nuclei in 3D Space
Source: PLoS Comput Biol. 2016 Jun 6;12(6):e1004970. doi: 10.1371/journal.pcbi.1004970 (PMC4894571; doi:10.1371/journal.pcbi.1004970)
Supplement: S2 Text — (DOCX) [file pcbi.1004970.s007.docx]

**S2 Text: procedure for calculation of principal curvatures of iso-intensity surfaces**

In differential geometry, curvatures at a point on a surface in 3D space are the curvatures of the intersection of the surface and the normal planes at the point. Maximum and minimum of the curvatures at the point are called as the principle curvatures $k_{1}$ and $k_{2}$, respectively. The Gaussian curvature $K$ and the mean curvature $H$ are the product and the mean of the principle curvatures, respectively. When the intensity of a 3D image at a point $x\in R^{3}$ is denoted by a function $f\left( x \right)=f\left( x_{1},x_{2},x_{3} \right)$, the iso-intensity surface with isovalue $I$ satisfies the implicit equation $f\left( x \right)=I$. Assuming that the 3D image is blurred by a Gaussian filter and then the function $f$ is continuous and differentiable, the Gaussian curvature $K$ and the mean curvature $H$ of the iso-intensity surface at a point $x$ can be obtained directly using the partial derivatives of $f$ at the point (without obtaining the iso-intensity surface) [1],

$$K=\frac{LN-M^{2}}{A},$$

$$H=\frac{EN-2FM+GL}{2A},$$

where

$${f_{x}}_{i}=\frac{\partial f}{\partial x_{i}}$$

$$f_{x_{i}x_{j}}=\frac{\partial^{2}f}{\partial x_{i}\partial x_{j}}$$

$$A=\frac{f_{x_{1}}^{2}+f_{x_{2}}^{2}+f_{x_{3}}^{2}}{f_{x_{3}}^{2}},$$

$$E=\frac{f_{x_{1}}^{2}+f_{x_{3}}^{2}}{f_{x_{3}}^{2}},$$

$$F=\frac{f_{x_{1}}f_{x_{2}}}{f_{x_{3}}^{2}},$$

$$G=\frac{f_{x_{2}}^{2}+f_{x_{3}}^{2}}{f_{x_{3}}^{2}},$$

$$L=\frac{2f_{x_{1}}f_{x_{3}}f_{x_{1}x_{3}}-f_{x_{1}}^{2}f_{x_{3}x_{3}}-f_{x_{3}}^{2}f_{x_{1}x_{1}}}{A^{\frac{1}{2}}f_{x_{3}}^{3}},$$

$$M=\frac{f_{x_{1}}f_{x_{3}}f_{x_{2}x_{3}}+f_{x_{2}}f_{x_{3}}f_{x_{1}x_{3}}-f_{x_{1}}f_{x_{2}}f_{x_{3}x_{3}}-f_{x_{3}}^{2}f_{x_{1}x_{2}}}{A^{\frac{1}{2}}f_{x_{3}}^{3}},$$

$$N=\frac{2f_{x_{2}}f_{x_{3}}f_{x_{2}x_{3}}-f_{x_{2}}^{2}f_{x_{3}x_{3}}-f_{x_{3}}^{2}f_{x_{2}x_{2}}}{A^{\frac{1}{2}}f_{x_{3}}^{3}}.$$

Then the principal curvatures can be obtained,

$$k_{1}=H+\left( H^{2}-K \right)^{\frac{1}{2}},$$

$$k_{2}=\frac{K}{k_{1}}.$$

We implemented these calculations as a Matlab code. Please note that the obtained curvatures are tolerant to noise because the Gaussian filter for blurring removes the noises from the image.

**Reference for S2 Text**

1. Thirion J-P, Gourdon A. Computing the Differential Characteristics of Isointensity Surfaces. Comput Vis Image Underst. 1995;61: 190–202. doi:10.1006/cviu.1995.1015
